# Overexpression of RASAL1 Indicates Poor Prognosis and Promotes Invasion of Ovarian Cancer

**DOI:** 10.1515/biol-2019-0015

**Published:** 2019-05-21

**Authors:** Rui-Xia Chang, Ai-Ling Cui, Lu Dong, Su-Ping Guan, Ling-Yan Jiang, Cong-Xiu Miao

**Affiliations:** 1 Department of Reproductive heredity, Heping Hospital affiliated to Changzhi Medical College, Changzhi, Shanxi 046000, P.R. China; 2 Department of gynecology, Heping Hospital affiliated to Changzhi Medical College, Changzhi, Shanxi 046000, P.R. China;; 3 Central Laboratory, Changzhi Medical College, Changzhi, Shanxi 046000, P.R. China; 4 Department of Hematological, Heping Hospital affiliated to Changzhi Medical College, Changzhi, Shanxi 046000, P.R. China; 5 Department of Information, Heping Hospital affiliated to Changzhi Medical College, Changzhi, Shanxi 046000, P.R. China

**Keywords:** RASAL1, ovarian cancer, poor prognosis, proliferation, invasion, migration, ERK

## Abstract

RAS protein activator like-1 (RASAL1) exists in numerous human tissues and has been commonly demonstrated to act as a tumor suppressor in several cancers. This study aimed to identify the functional characteristics of RASAL1 in ovarian adenocarcinoma and a potential mechanism of action. We analyzed RASAL1 gene expression in ovarian adenocarcinoma samples and normal samples gained from the GEO and Oncomine databases respectively. Then the relationship between RASAL1 expression and overall survival (OS) was assessed using the Kaplan-Meier method. Furthermore, the biological effect of RASAL1 in ovarian adenocarcinoma cell lines was assessed by Quantitative real time-PCR (qRT-PCR), Cell Counting Kit-8 (CCK-8), western blot, wound healing and transwell assay. The statistical analysis showed patients with higher RASAL1 expression correlated with worse OS. The in vitro assays suggested knockdown of RASAL1 could inhibit cell proliferation, cell invasion and migration of ovarian adenocarcinoma. Moreover, the key proteins in the mitogen-activated protein kinase/extracellular signal-regulated kinase (MEK/ERK) signaling pathway were also decreased in ovarian adenocarcinoma cells with RASAL1 silencing. These findings provide promising evidence that RASAL1 may be not only a powerful biomarker but also an effective therapeutic target of ovarian adenocarcinoma.

## 1 Introduction

Ovarian cancer is ranked as the fifth most common type of cancer in women and the fourth most common cause of cancer death in women worldwide. The American Cancer Society estimates over 20 000 new cases of ovarian cancer will be diagnosed each year and almost 15 500 women died of this disease [1, 2]. Most ovarian cancer cases are diagnosed as advanced stages (III and IV) and the 5-year survival is <30% [3, 4]. Although improvements have been made in conventional treatment approaches, such as surgery and chemotherapy, survival rates have shown relatively little improvement over the past decade [5, 6]. The majority of patients with advanced ovarian cancer experience relapse and eventually succumb to ovarian cancer. Thus, new therapeutic strategies are urgently required.

RASAL1, encoding a member of RAS-GAP, can hydrolyze GTP to GDP and inactivate the Ras protein, thereby participating in many cellular processes, including cell proliferation, differentiation and apoptosis [7, 8]. RASAL1, has recently been identified as an important tumor suppressor for numerous cancers [8, 9]. Liu et al. identified RASAL1 as a major tumor suppressor that is frequently inactivated by hypermethylation and mutations for thyroid cancer [10]. Reductions in RASAL1 expression were detected more frequently in advanced lesions than in small adenomas, suggesting that RASAL1 functions in the progression of benign colonic neoplasms [11]. Activated RASAL1 pathway could inhibit tumor growth by directly targeting the RASAL1 3’-UTR in sorafenib-sensitized hepatocarcinoma cells [7]. RAS proteins have been reported to participate in ovarian cancer progression through different pathways. Van et al. reported miR-634 restores drug sensitivity in resistant ovarian cancer cells by targeting the Ras-MAPK pathway [12]. Bauckman et al. reported iron modulates cell survival in a Ras-dependent manner in ovarian cells [13]. On the contrary, RASAL overexpression is observed in thyroid cancer cells in contrast with non-cancer cells [14]. Of note, it is also mentioned that RASAL1 overexpression was associated with frequent occurrence of distant metastases. Longer overall survival (OS) times are reported for patients without RASAL1 expression as well as lower frequencies of distant metastases in oesophagogastric adenocarcinoma [15]. As of yet, the relationship between RASAL1 and ovarian cancer has not been unambiguously determined.

To gain insight into the effect of RASAL1 inhibition on ovarian cancer progression, we evaluated the effect of RASAL1 on tumor growth using bioinformatics analysis and in vitro experiments respectively. In the bioinformatics analysis, we determined the clinical implications between RASAL1 expression and prognosis of ovarian cancer. We further assessed the biological effect and mechanism of action of RASAL1 on human ovarian cancer cell lines.

## 2 Methods

### 2.1 Gene database analysis

Differences in RASAL1 gene expression between 12 normal samples and 12 ovarian adenocarcinoma samples from the GEO database were analyzed. RASAL1 gene expression differences were also analyzed for 10 peritoneum samples and 43 ovarian serous adenocarcinoma samples obtained from oncomine database. OS was analyzed based on the samples obtained from the TCGA database using Perl’s language package.

### 2.2 Cell culture

Human ovarian adenocarcinoma cells HEY were purchased from the Cell Bank of Type Culture Collection of Chinese Academy of Sciences (Shanghai, China). Human ovarian adenocarcinoma cells A2780 and human normal ovarian cells IOSE80 were purchased from the American Type Culture Collection (ATCC, USA). Cells were cultured with Roswell Park Memorial Institute-1640 (RPMI-1640; Gibco, USA) medium at 37°C with an atmosphere of 5% CO_2_ in a humidified cell incubator. The RPMI-1640 medium contains 10% fetal bovine serum (FBS), 0.1 mg/ml streptomycin (Sigma, USA), 100 U/ml penicillin (Sigma, USA). Then the cells were digested with 0.25% trypsin (Solarbio, Beijing), as growing to the logarithmic growth phase, terminated by re-adding culture medium. Cells were resuspended to single cell suspensions and seeded into a 6-well plate for the following experiments.

### 2.3 Cell transfection

HEY cells were digested with trypsin until the mid-log phase and then seeded into 6-well plates (Sigma, USA) with a density of 1×10^5^ cells per well. After 8 h of cell adherence to the flask, 10 μl small interference- (si-)RNA or si-conRNA and 5 μl Turbofect^TM^ in vitro Transfection Reagent (Thermo Scientific, St.Leon-Rot, Germany) were gently mixed for 20 min. The HEY cells were incubated with this complex in serum-free RPMI-1640 medium for 6 hours at 37°C. Next, the cells were incubated at 37°C with RPMI-1640 medium for 48 h, and cells were collected to prepare for miRNA and protein quantification. Si-RNAs targeting RASAL1 were: si-RASAL1#1: 5’- AACAGGGCAGAAAGUUGUA -3’ (forward), si-RASAL1#2: 5’ - AUGAAAUGGGAUCAAGUGG -3’ (forward).

### 2.4 Quantitative real time-PCR (qRT-PCR)

Total RNA was extracted using TRIzol reagent (Invitrogen, USA) according to the instruction manual. To quantify RASAL1 gene expression, total RNA was first reverse transcribed using mRNA first-Strand cDNA synthesis kit (Takara, Dalian), followed by qRT-PCR using 1 μg cDNA. The steps for the qRT-PCR program were: 95°C for 5 min, followed by 40 amplification cycles (95°C for 30 sec, 60°C for 45 sec) and 72°C for 30 min. Primers (Sangon, Shanghai) used for RASAL1 were: 5’- CAGGGACCTCGTTAATGGCT -3’ (forward) and 5’- CCGTTCAGACAGCTGACGTT -3’(reverse). GAPDH was used as an internal control, and 5 replicates were performed for every sample. Primers (Sangon, Shanghai) used for GAPDH were: 5’- GGAGCGAGATCCCTCCAAAAT -3’ (forward) and 5’- GGCTGTTGTCATACTTCTCATGG -3’ (reverse). The 2^-ΔΔCt^ method was used to analyze relative quantification of RASAL1, with 3 independent repeats.

### 2.5 Western blot

After the cells were transfected for 72 h, cells were treated with radio immunoprecipitation assay (RIPA; Cwebio, Beijing) lysis buffer and the concentration of RASAL1 was tested using a BCA kit (Cwebio). Extracted proteins (20 μg) were separated in polyacrylamide gel and then transferred to a polyvinylidene fluoride (PVDF; Thermo, USA) membrane. Then the membrane was blocked with 5% non-fat milk for 1 h and incubated with primary antibodies overnight at 4°C. The membrane was washed for 5 min with PBS three times and incubated with horseradish peroxidase (HRP)-conjugated secondary antibody (1: 5000, PTG, USA) for 1 h. After the final washing, the signal was enhanced using ECL reagent (PTG, USA) quantitated by Quantity One software (Bio-Rad Laboratories, USA), with GAPDH as the internal control. Western blots were carried out with following primary antibodies: RASAL1, mitogen-activated protein kinase (MEK), ERK (extracellular signal-regulated kinase) and phosphorylated-(p-)MEK, p-ERK (dilution at 1:1,000, Cell Signaling Technology, USA), and GAPDH (1: 10000, PTG, USA).

### 2.6 Cell proliferation assay

After the cells were transfected with siRNA or si-conRNA for 72 h, 100 μl of HEY cell suspension containing 1×10^3^ cells was routinely seeded in a 96-well plate. Then the 96-well plate was placed in carbon dioxide incubator for 96 h. During the incubation, the cells were detected every 24 h and pretreated with 10 μl CCK8 reagent (Solarbio). Cell proliferation followed a curve based on absorbance measurements (450 nm).

### 2.7 Invasion and migration assay

Transwell invasion assay was performed in 24-well transwell plate. 100 μl Matrigel matrix gel (BD Bioscience, USA), diluted in serum-free medium (1: 6) overnight, was added to the insert chamber of a 24-well plate. The 24-well plate was then cultured at 37°C for 4-6 h in a carbon dioxide incubator. A total of 1x10^5^ transfected cells suspended in 100 μl serum-free RPMI 1640 medium were then seeded into the insert chamber for 24 h of incubation, with lower chambers filled with 500 μL RPMI 1640 medium with 10% FBS. After 72 h of incubation, non-invading cells in the upper chambers were removed by scrubbing gently with a cotton-tipped swab. The invading cells were fixed with 4% paraformaldehyde for 30 min, and then stained with 0.1% crystal violet (MCE China, Shanghai, China) for 20 min at room temperature. After washing with PBS, the invading cells were imaged by five random fields for each insert, and the mean number was calculated as per field.

The migration experiment procedure is similar to the invasion assay, with the difference being that the transwell chamber is not required to be paved with Matrigel matrix.

### 2.8 Wound healing assay

The migration of HEY cells in the siRNA or si-conRNA group was determined by a wound healing assay. Briefly, 5×10^5^ cells/well were cultured in 24-well plates in triplicate, and when they reached nearly 90% confluence, the cells were scratched with a tip across the wells. The cells were continually cultured for 24h and imaged using ImageJ software (NIH, Rockville, USA).

### 2.9 Statistical analysis

SPSS 22.0 statistical analysis software was used to analyze the experimental data. The OS was analyzed using the Kaplan-Meier method and Log-rank test. The differences between groups were analyzed using one-way analysis of variance (ANOVA) with post-hoc Dunnett analysis. Measurement data was expressed as mean ± standard (Mean ± SD), between the two groups using a t test. *P* < 0.01 was statistically significant.

## 3 Results

### 3.1 RASAL1 gene expression in ovarian adenocarcinoma samples

**Figure 1:**
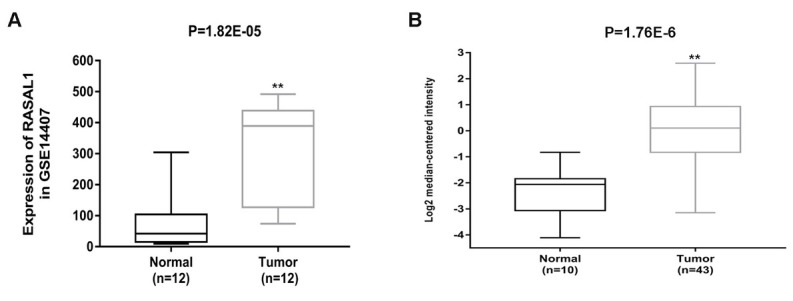
RASAL1 gene expression differences between normal samples and ovarian adenocarcinoma samples (A) RASAL1 gene expression differences between 12 normal samples and 12 ovarian adenocarcinoma samples gained from GEO. **P < 0.01 compared with normal samples. (B) RASAL1 gene expression differences between 10 peritoneum samples and 43 ovarian serous adenocarcinoma samples. **P < 0.01 compared with normal samples.

We first calculated RASAL1 gene expression differences between 12 normal samples and 12 ovarian adenocarcinoma samples obtained from the GEO database. Then we analyzed RASAL1 gene expression differences between 10 peritoneum samples and 43 ovarian serous adenocarcinoma samples obtained from the oncomine database. As shown in Fig 1, RASAL1 expression levels were significantly upregulated in ovarian adenocarcinoma samples (both *P* < 0.01).

**Figure 2:**
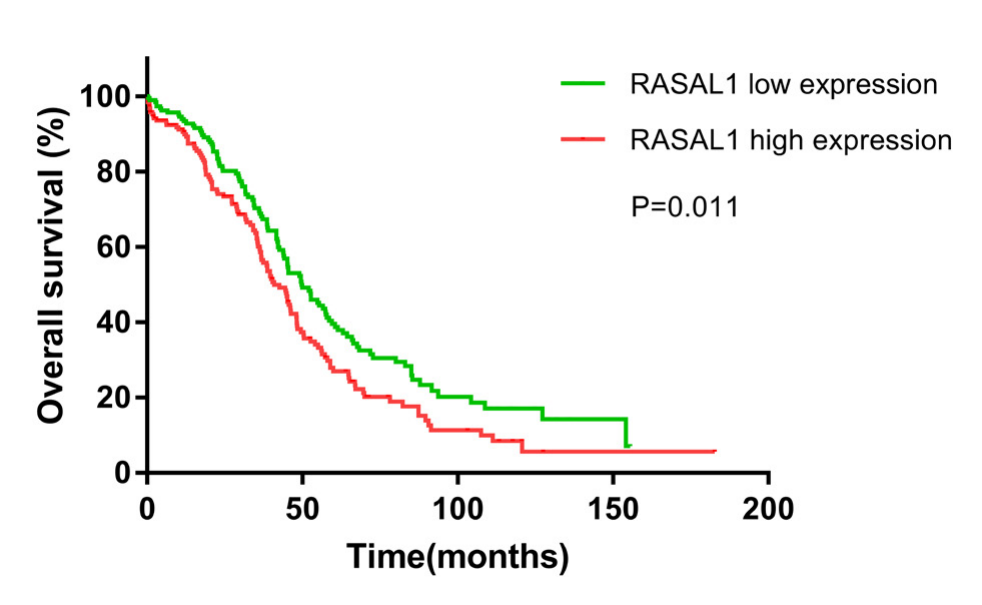
The overall survival (OS) based on the RASAL1 expression level.

The OS based on the RASAL1 expression level was further analyzed using publicly available data of 377 ovarian adenocarcinoma patients obtained from the TCGA database. As shown in Fig 2, higher RASAL1 expression correlated with worse OS (*P* < 0.01).

### 3.2 Expression of RASAL1 in ovarian adenocarcinoma cell lines

**Figure 3:**
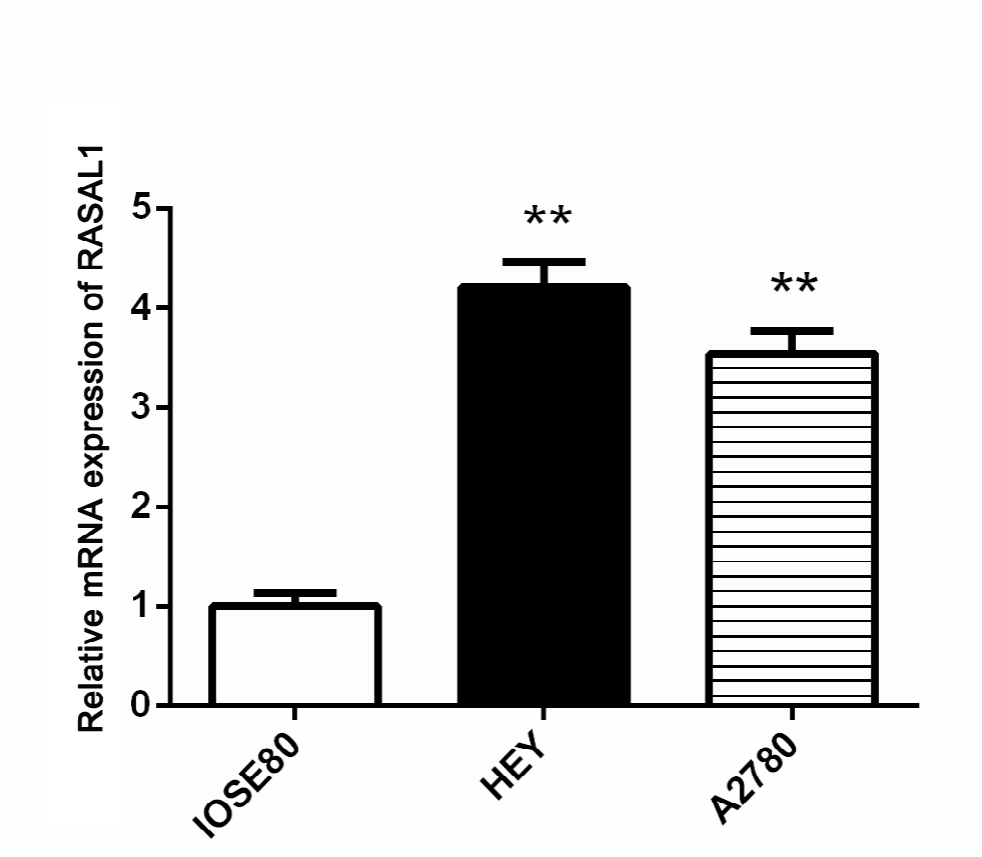
Analysis of RASAL1 expression levels in 2 ovarian adenocarcinoma cell lines (HEY and A2780) compared with normal human ovarian adenocarcinoma cell IOSE80 using qRT-PCR. **P < 0.01 compared with IOSE80.

To investigate the biological functional of RASAL1 in ovarian adenocarcinoma cells, we measured RASAL1 gene expression in ovarian adenocarcinoma cell lines (HEY and A2780) and normal human ovarian adenocarcinoma cells IOSE80 by qRT-PCR. As shown in Fig 3, RASAL1 expression levels in ovarian adenocarcinoma cell lines (HEY and A2780) were significantly higher (both *P* < 0.01) compared to the normal cell line, and RASAL1 expression levels in the HEY cell line was much higher than in the A2780 cell line (*P* < 0.01). Therefore, we selected the HEY cell line as the ovarian adenocarcinoma cell model for the following tests.

### 3.3 Knockdown of RASAL1 in ovarian adenocarcinoma cell line HEY

**Figure 4:**
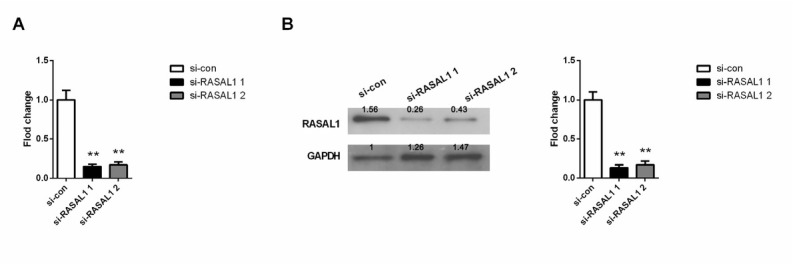
The relative expression levels of RASAL1 in HEY cells transfected with si-con or si- RASAL1 (si- RASAL11 1# and si- RASAL1 2#) were measured using qPCR and western blot. (A) Relative RASAL1 gene expression measured using qPCR. (B) Relative RASAL1 protein expression measured using western blot. si-con, negative control. **P < 0.01 compared with si-con group.

We synthesized two different siRNAs to silence endogenous RASAL1 in HEY cells. After 72 hours of transfection, RASAL1 expression was analyzed by qRT-PCR (result shown in Fig 4A) RASAL1 protein expression was also measured by western blot and the result is showed in Fig 4B The results of qRT-PCR and western blot analysis indicated the knockout efficiency of siRNA2 and siRNA1 reached 80%, with siRNA1 being more efficient. Thus, we selected siRNA1 for the following tests.

### 3.4 Proliferation of cell line HEY with knockdown of RASAL1

**Figure 5:**
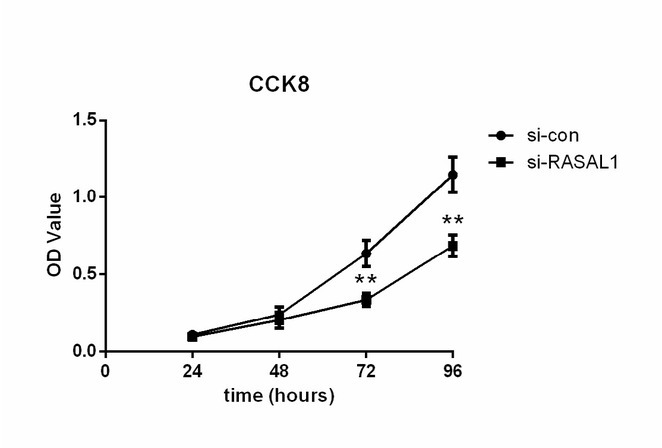
The proliferation of ovarian adenocarcinoma cell HEY transfected with RASAL1 was inhibited. si-con, negative control. **P < 0.01 compared with si-con group.

Subsequently we conducted CCK8 assays to demonstrate the proliferation of ovarian adenocarcinoma cell line HEY transfected with siRNA1. As shown in Fig 5, we found knockdown of RASAL1 expression significantly attenuated the cell viability rate in HEY cells. In other words, knockdown of RASAL1 could significantly inhibit cell proliferation for HEY cells.

### 3.5 Migration and invasion of cell line HEY with knockdown of RASAL1

**Figure 6:**
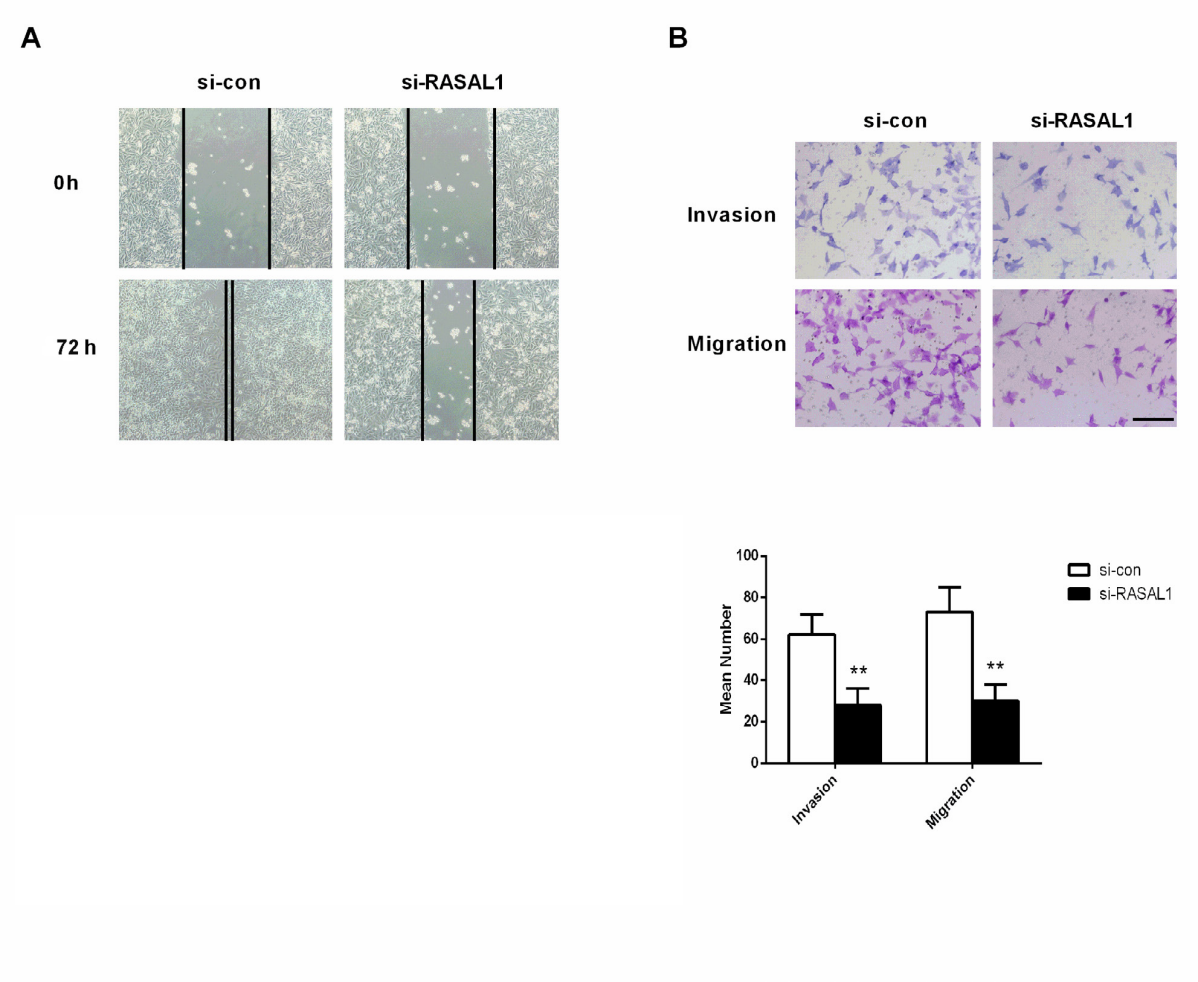
The migration and invasion of ovarian adenocarcinoma cell line HEY with knockdown of RASAL1 was inhibited. (A) Wound healing assay was conducted 72 h after transfection to determine the migration ability of HEY cells. (B) Transwell assay was conducted 72 h after transfection to determine the invasion and migration ability of HEY cells. si-con, negative control, **P < 0.01 compared with si-con group.

Given that silencing of RASAL1 inhibits the proliferation of ovarian adenocarcinoma cell line HEY, we then investigated cell migration and invasion in HEY transfected with si-RASAL1 using a wound healing assay and a transwell assay. The results of the wound healing assay showed that HEY cells transfected with RASAL1 had significantly inhibited and delayed closure compared to the control group (Fig. 6A) The data from the transwell assay showed that the invaded number of HEY cells transfected with RASAL1 (28 ± 8) was significantly reduced compared to the control group (62 ± 9) (*P* < 0.01, Fig. 6B, 6C) The migrated number of HEY cells transfected with RASAL1 (30 ± 6) was also significantly decreased compared with the control group (72 ± 13) (*P* < 0.01, Fig. 6B and C) These results suggested that knockdown of RASAL1 could significantly inhibit cell migration and invasion capabilities for HEY cells.

### 3.6 Knockdown of RASAL1 inhibits MAPK signaling pathway in ovarian adenocarcinoma

**Figure 7:**
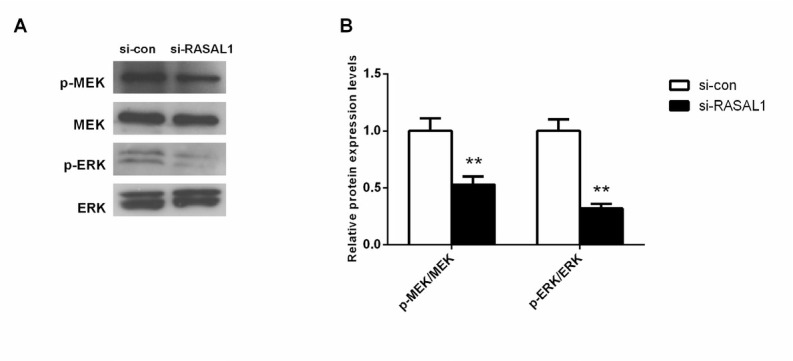
Knockdown of RASAL1 inhibits the ERK signaling pathway in HEY cells. Expression level of proteins was measured in the HEY cells after 72 h transfection using western blot. si-con, negative control. **P < 0.01 compared with si-con group.

Previous studies have shown that ERK holds an important effect in the MAPK signaling pathway which promotes cell growth. To determine whether RASAL1 manipulates ovarian adenocarcinoma progression, we evaluated the activation of key signaling proteins in the MAPK signaling pathway, including p-ERK and p-MEK. We found that HEY cells with knockdown of RASAL1 had lower expression of p-ERK and p-MEK (Fig. 7). Accordingly, deletion of RASAL1 induced a significant reduction of the ERK signaling pathway in the ovarian adenocarcinoma cell line HEY.

## 4 Discussion

In the present study, we first found that RASAL1 was overexpressed both in ovarian adenocarcinoma tumor samples and cell lines. Next, we found overexpression of RASAL1 was determined to be largely associated with lower overall survival in ovarian adenocarcinoma samples. Further, knockdown of RASAL1 could significantly impede ovarian adenocarcinoma cell proliferation, invasion, migration, which might work through the MAPK signaling pathway.

The Ras gene encodes proteins that mediate growth factors, cytokines and multiple extracellular signals which have crucial effects in cell proliferation, differentiation and apoptosis [16]. With the Ras gene abnormally activated, its encoded proteins maintain an activated state continuously and activate downstream signaling molecules, resulting in uncontrolled cell growth and unlimited proliferation, which in turn leads to tumors [17, 18]. As mentioned in previous studies, RAS over expression is correlated with poor survival in ovarian cancer [19, 20, 21]. RAS mutation coupled with p53 deletion results in ovarian carcinosarcomas with high grade and poorly differentiated in mice [22]. RASAL1 is a recently discovered gene located on chromosome 12, which encodes a protein that activates the RasGTP enzyme, which inactivates Ras by converting GTP and to GDP, and thus participates in cell proliferation, differentiation and apoptosis [23, 24, 25]. Based on this mechanism, lower expression of RASAL1 should be found in tumor samples as up-regulated RASAL1 suppresses tumor progression. Accordingly, down-regulated RASAL1 expression was detected more frequently in advanced lesions than in small adenomas, suggesting that RASAL1 functions in the progression of benign colonic neoplasms [11]. In contrast, RASAL expression was found up-regulated in thyroid cancer cells compared with normal tissues [14]. Furthermore, Knief et al demonstrated that increased RASAL1 expression was correlated with more frequent occurrences of distant metastases. Patients with decreased RASAL1 expression had significantly longer overall survival times, as well as lower frequencies of distant metastases in oesophagogastric adenocarcinoma [15]. These conflicting studies have implications in elucidating novel insight as to how RASAL1 affects cancer occurrence and development, including ovarian adenocarcinoma. In this work, our observations showed that RASAL1 expression is increased in ovarian adenocarcinoma tumor samples compared with normal samples. Furthermore, we found that up-regulated RASAL1 expression suggests a worse OS of ovarian adenocarcinoma tumor patients. Similarly, RASAL2 is functionally equivalent with RASAL1 upregulation and gene copy number gain which is also found in high-grade serous ovarian cancer, and has recently been found to share a similar molecular portrait to TNBC through large-scale genomic analyses [26]. RASAL2 also activates RAC1 to promote triple-negative breast cancer progression [27]. This suggests that a probable oncogenic role of RASAL1 is possible. The contradictory roles of RASAL1 in ovarian cancer underscore an important contextual dependency of RASAL1 function in human cancers.

In vitro knockdown of RASAL1 demonstrated the oncogenic role of RASAL1 in ovarian cancer invasion and metastasis. In terms of mechanism, we showed that the oncogenic function of RASAL1 in ovarian cancer also influenced MAPK signaling as RASAL1 depletion attenuates the activity of key proteins p-ERK and p-MEK in ovarian cancer cells HEY. The ability of RASAL1 to suppress RAS-coupled MAPK pathways is owing to its function as a classical RasGAP [28]. Consistent with previous reports, we also found ovarian adenocarcinoma cell invasion and migration was inhibited accompanied by inhibition of cell proliferation with RASAL1 gene knockdown. The MAPK signaling pathways are important for tumor migration and invasion. The EGFR-MEK-ERK signaling pathway has been reported to mediate ovarian adenocarcinoma cell motility and invasiveness [29]. P-MEK and p-ERK are two vital activated protein kinases in the MAPK signaling pathway [30]. The protein levels of p-MEK and p-ERK were also tested by western blot. Our results indicate that RASAL1 is involved in the regulation of MAPK signaling pathways, probably resulting in inhibition of ovarian adenocarcinoma cell proliferation.

Collectively, in this study, we clarified that RASAL1 was increased in ovarian adenocarcinoma tumorous tissues and HEY cells, which correlated with poor prognosis in ovarian adenocarcinoma patients. Experiments showed RASAL1 deletion repressed ovarian adenocarcinoma cell proliferation, migration and invasion. Our findings imply that RASAL1 could function as an important factor in promoting oncogenesis for ovarian adenocarcinoma.

## Abbreviations

RASAL1: RAS protein activator like-1
OS: overall survival
SI: small interference
MEK: mitogen-activated protein kinase
p-MEK: phosphorylated-mitogen-activated protein kinase
ERK: extracellular signal-regulated kinase
p-ERK: phosphorylated- extracellular signal-regulated kinase
